# Granulocyte Colony Stimulating Factor (G‐CSF) and Olfactory Function—A Clinical Pilot Study

**DOI:** 10.1002/lio2.70143

**Published:** 2025-06-13

**Authors:** C. A. Hintschich, K. Resler, C. Brückner, A. Altundag, K. Trautmann, K. Hölig, F. Kroschinsky, M. Pieniak, T. Hummel

**Affiliations:** ^1^ Smell & Taste Clinic, Department of Otorhinolaryngology TU Dresden Dresden Germany; ^2^ Department of Otorhinolaryngology University of Regensburg Regensburg Germany; ^3^ Department and Clinic of Otolaryngology, Head and Neck Surgery Wroclaw Medical University Wroclaw Poland; ^4^ Department of Otorhinolaryngology Biruni University Medical Faculty Istanbul Turkey; ^5^ Medical Department I TU Dresden Dresden Germany; ^6^ Institute of Psychology, University of Wroclaw Wroclaw Poland

**Keywords:** olfaction, smell, stem cells

## Abstract

**Background:**

Although the olfactory epithelium, including its neuronal cell line, has inherent regenerative potential, therapeutic options remain limited. Promising effects of granulocyte colony stimulating factor (G‐CSF) on olfactory regeneration have been observed in both animal and human studies. In this study, we assessed olfaction before and after G‐CSF administration in myeloma patients who underwent autologous stem cell transplantation and in allogeneic stem cell donors.

**Methods:**

A total of 40 subjects were included in this study (10 myeloma patients, 10 allogeneic stem cell donors, 20 controls who did not receive any G‐CSF). Olfactory function was psychophysically assessed using the threshold and extended identification domain of the Sniffin' Sticks test.

**Results:**

After G‐CSF administration, threshold scores were slightly enhanced in both myeloma patients (8.9 ± 3.6 vs. 9.3 ± 3.3) and allogeneic stem cell donors (10.2 ± 3.5 vs. 11.8 ± 2.9). However, this effect was not statistically significant. For olfactory identification, no improvement was observed.

**Conclusion:**

Even though being not statistically significant, the findings of this study align with previous evidence and underline the potential of G‐CSF on olfactory regeneration. However, additional studies, including carefully designed animal trials, are required to comprehensively evaluate this promising therapeutic option.

**Level of Evidence:** 2.

## Graphical abstract

Promising effects of granulocyte colony stimulating factor (G‐CSF) on olfactory regeneration have been observed in both animal and human studies. Even though being not statistically significant, our study underlines the potential of G‐CSF on olfactory regeneration. However, carefully designed animal trials are required to comprehensively evaluate this promising therapeutic option.

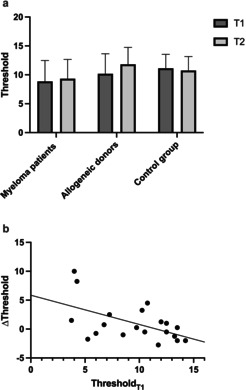

## 1 Introduction

Olfaction helps to detect and locate potential environmental hazards [1]. This applies to toxic gases, burnt food, fires, as well as for verifying the quality of food. Moreover, the sense of smell provides retronasal flavor, stimulates dietary behavior, and influences interpersonal relations [2, 3, 4, 5]. Consequently, its impairment places individuals at risk of critical situations, such as being unable to respond adequately to fires or incidents of food contamination. Beyond this, compromised olfactory function profoundly affects psychological well‐being and quality of life [6]. While various causes can underlie chronic olfactory dysfunctions, the prevalent etiologies, including aging, chronic rhinosinusitis, and post‐COVID‐19 sequelae [7], are partly due to a reduced number of olfactory sensory neurons [8, 9].

However, unlike most other senses, the olfactory mucosa possesses a unique regenerative potential [10]. This distinct ability is due to two distinct populations of stem cells: Globose basal cells (GBCs) function as active stem cells to maintain the olfactory epithelium, differentiating into various cell types of the olfactory epithelium, including olfactory sensory neurons. In contrast, horizontal basal cells represent a stem cell reserve and can only be hardly activated [11]. This capacity for olfactory regeneration has been studied through different approaches, such as psychophysical [12], immunohistopathological [13], and cell culture studies [14]. Despite this inherent regenerative capability, the therapeutic options are limited [15, 16]. Consequently, there exists a necessity for pharmaceutical agents that can support this regenerative capacity.

Granulocyte colony stimulating factor (G‐CSF) is a promising candidate for such a drug. This hematopoietic cytokine does not only promote the survival, proliferation, and differentiation of neutrophil cells, but also has neuroprotective and neuroregenerative effects [17, 18]. Its DNA‐recombinant *Filgrastim* is used as a myeloid growth factor for the treatment of severe neutropenia due to chemotherapy, radiation, or HIV for 30 years now. Additionally, Filgrastim is used in the preparation of peripheral stem cell donations to mobilize stem cells out of the bone marrow to the peripheral blood.

A therapeutic potential of G‐CSF on the olfactory epithelium was first shown in 2010 [19]. In this rodent study, 1 month following the allogenic transplantation of GFP‐positive bone marrow cells, the olfactory epithelium of the host mice was damaged using methimazole. Subsequently, following subcutaneous administration of G‐CSF, a significantly enhanced engraftment of GFP‐positive stem cells into the previously injured olfactory epithelium was observed, as compared to the control group.

Moreover, a positive effect of G‐CSF on human olfactory function was observed in patients with Barth syndrome [20]. In addition to growth delay, cardiac and skeletal myopathy, this rare X‐linked recessive condition is frequently associated with neutropenia necessitating G‐CSF treatment whereas olfaction is not compromised. The study revealed that those patients with Barth syndrome, who underwent application of recombinant G‐CSF to treat their severe neutropenia scored higher in all domains of the Sniffin' Sticks smell test than a group of patients with Barth syndrome who have not received any G‐CSF. However, the difference was statistically not significant—potentially due to the small study population.

These findings are in good agreement with G‐CSF serum levels in early Alzheimer's disease: Olfactory dysfunction is often the very first symptom of Alzheimer's disease [21]. Correspondingly, individuals with early Alzheimer's disease exhibit significantly lower serum G‐CSF levels compared to healthy controls [22].

The above evidence suggests a potential role of G‐CSF in olfactory regeneration. We therefore hypothesized that the administration of G‐CSF improves the olfactory function.

## 2 Materials and Methods

This prospective study was conducted at TU Dresden between February 1, 2019 and April 31, 2021, at the Smell and Taste Clinic of the Department of Otorhinolaryngology in collaboration with the Department of Internal Medicine. Following prior approval from the local ethics committee (reference: EK288072018) the study was conducted in accordance with the ethical standards of the Declaration of Helsinki and its later amendments. Comprehensive information was given to the patient during an interview and in written form, and their consent was likewise procured within this interview and in written form.

### 2.1 Study Design

The cohort consisted of three groups: One group comprised patients with myeloma who had undergone prior induction chemotherapy and were subsequently administered G‐CSF for steady‐state stem cell mobilization. A second study group consisted of healthy stem cell donors who received G‐CSF treatment prior to allogenic stem cell donation. In both study groups, G‐CSF was administered on 5 consecutive days (subcutaneous G‐CSF at a dose of 10 μg/kg body weight). A control group of healthy adults did not receive any G‐CSF treatment. Exclusion criteria encompassed age below 18 or above 75 years, significant comorbidities or previous surgery of the nose or paranasal sinuses, Alzheimer's or Parkinson's disease, other relevant pre‐existing neurological diseases or cognitive impairment, and a recent infection with SARS‐CoV‐2.

For both study groups, the initial psychophysical assessment of olfaction was conducted prior to the G‐CSF treatment. The second assessment took place 4 weeks after stem cell mobilization.

### 2.2 Assessment of Olfactory Function

Olfactory tests were performed birhinally using the validated Sniffin' Sticks test (SST; Burghart Medizintechnik, Holm, Germany) [23]. In this study the subtests for threshold (T) and the extended identification (I) subtest were used, which has been previously described in detail [24, 25]. The two test scores were added up to one TI score which had a maximum value of 48.

### 2.3 Statistical Analyses

Statistical analyses were performed using SPSS software (Statistical Packages for Social Sciences, Version 26.0; IBM, Chicago, IL, USA). Descriptive statistics included means and standard deviations. Continuous data were tested for statistical significance using Student's *T*‐test or analysis of variance (ANOVA) with post hoc Bonferroni corrections. Categorical data were compared between groups using chi‐square tests. The *α* level was defined as 0.05.

## 3 Results

A total of 40 subjects were included in the study. Among them, 10 were myeloma patients, 10 were allogeneic stem cell donors, and 20 were healthy controls. The gender distribution was balanced across the groups (*χ* ^2^(2) = 0.22, *p* = 0.90; Table 1). However, myeloma patients were older than controls and allogeneic donors (*F*(2.39) = 10.44, *p* < 0.001; Table 1).

**TABLE 1:** Demographic data and results of psychophysical testing of olfaction; a, b, c, d, e, f: *p* > 0.05.

	Myeloma patients	Allogeneic donors	Control group	*p*
*n*	10	10	20	
Female	40%	30%	35%	*χ* ^2^ = 0.22, 0.90
Age (years)	60.2 ± 5.8	34.1 ± 10.9	47.3 ± 15.7	< 0.001
Threshold_T1_	8.9 ± 3.6^a^	10.2 ± 3.5^c^	11.1 ± 2.4^e^	0.16
Threshold_T2_	9.3 ± 3.3^a^	11.8 ± 2.9^c^	10.7 ± 2.4^e^	0.15
Identification_T1_	24.9 ± 3.4^b^	27.0 ± 1.6^d^	26.0 ± 3.4^f^	0.32
Identification_T2_	24.6 ± 3.8^b^	26.9 ± 2.2^d^	26.3 ± 3.0^f^	0.21

At T1 neither olfactory threshold score (*p* = 0.16) nor the score for olfactory identification differed between the three groups (*p* = 0.32; Table 1). Interestingly, only two patients were hyposmic when using the cutoff values for the TI test [26].

**FIGURE 1:**
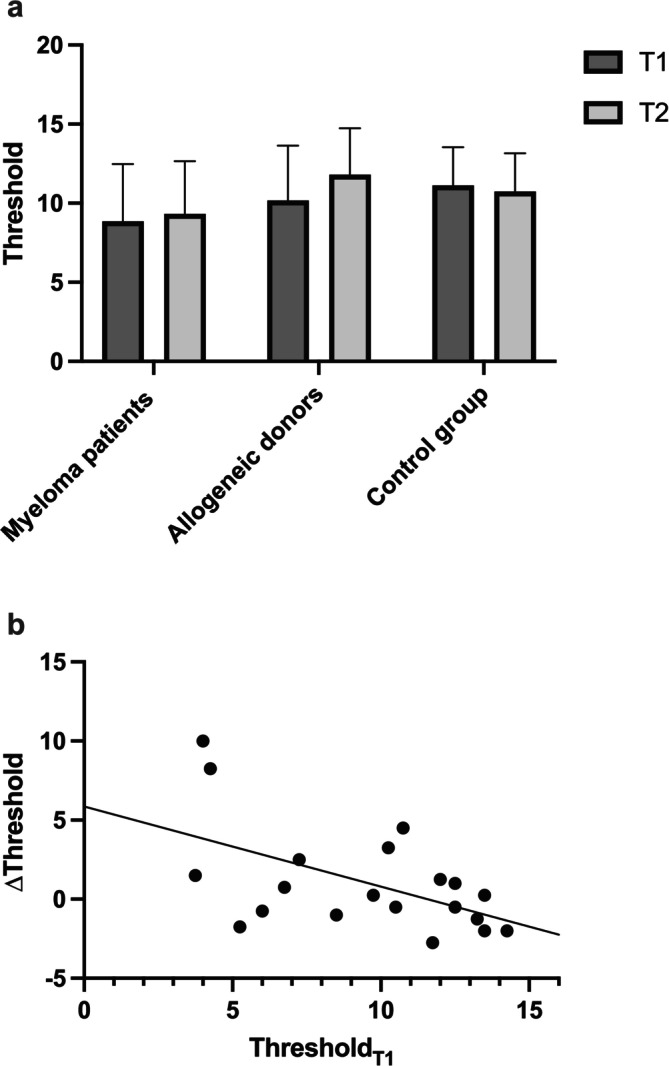
(a)Olfactory threshold score enhances in both G‐CSF groups compared to controls; (b) Negative correlation between the threshold score at T1 and ∆Threshold in subjects who received G‐CSF.

In mean 30.1 days (myeloma: 28.2 days; allogeneic: 31.9 days; *p* = 0.76) after the last G‐CSF administration, psychophysical assessment was repeated and showed a slightly enhanced threshold score for both study groups compared to T1 (myeloma: 8.9 ± 3.6 vs. 9.3 ± 3.3; allogeneic: 10.2 ± 3.5 vs. 11.8 ± 2.9; Figure 1; Table 1). However, this effect was not statistically significant (*p* = 0.69 and 0.13). For the identification test, no such improvement was observed after G‐CSF (Table 1).

However, for G‐CSF treated individuals (myeloma patients and allogeneic donors) there was a negative correlation between baseline olfactory threshold and the change of threshold (∆Threshold; *r* = −0.53; *p* = 0.015; Figure 1b). This could not be observed for the control group (*r* = −0.31; *p* = 0.19).

## 4 Discussion

This study did not show a significant improvement in olfactory threshold after G‐CSF administration; however, a tendency toward ent was observed. Thus, these findings are consistent with a previous study in which Barth syndrome patients who received G‐CSF reached higher scores in different psychophysical tests, including the olfactory threshold score [20]. Interestingly, we further observed that the improvement in olfaction was dependent on the olfactory function level before the G‐CSF administration. Patients with an initially lower threshold score were more likely to experience an enhancement in the threshold score. Furthermore, no improvement was observed in the control group, supporting the assertion that the enhancement in olfactory threshold in the G‐CSF groups is not due to a learning effect.

The potential effect of G‐CSF may arise from the recruitment of multipotent bone marrow stem cells into the olfactory epithelium from the bloodstream. Circulating stem cells are known to be attracted to damaged tissue [27]. This phenomenon has been investigated in the context of the olfactory epithelium using a mouse model. In this model, it was demonstrated that allogeneic stem cells labeled with GFP successfully integrated into the previously damaged olfactory epithelium [19]. Additionally, co‐labeling with GFP, cytokeratin, GAP43, and OMP revealed that once engrafted, these allogeneic stem cells underwent further differentiation into various cell types associated with the neuronal lineage of olfactory cells, including GBCs, immature neurons, and mature olfactory neurons. As G‐CSF is known to mobilize bone marrow stem cells into circulation [17], it is proposed that this regenerative effect could potentially lead to the secondary regeneration of the olfactory epithelium and, consequently, the restoration of olfactory function.

However, this study has some limitations. (i) The cohort was relatively small, with only 10 patients in both study groups. Hence, given the limited sample size, a statistically significant result at the *α*level of 0.05 would have required a larger effect size than observed in the normosmic population. (ii) The very majority of patients in both study groups had normosmia. Hence, in those patients, a potential improvement of the olfactory function is possible but not very likely compared to hyposmic patients. This is supported by previous work on olfactory training [28]. (iii) The patients did not experience acute damage to the olfactory epithelium as performed in the mouse study by Nishizaki et al. [19]. Hence, the inflammatory stimulus to attract circulating stem cells to engraft in the olfactory epithelium might have been missing.

## 5 Conclusion

This study supports previous publications on a potential regenerative effect on olfaction through G‐CSF. We could observe that both the testing scores for olfactory threshold (T) increased in both study groups that had received G‐CSF. However, the effect was not significant—presumably because of the small size of the cohort. Hence, further studies—including well‐designed animal trials—should further assess this promising therapeutic approach.

## Conflicts of Interest

The authors declare no conflicts of interest.
